# Optimization-Based Wi-Fi Radio Map Construction for Indoor Positioning Using Only Smart Phones

**DOI:** 10.3390/s18093095

**Published:** 2018-09-14

**Authors:** Jian Tan, Xiangtao Fan, Shenghua Wang, Yingchao Ren

**Affiliations:** 1 Hainan Key Laboratory of Earth Observation, Sanya 572029, China; fanxiangtao_radi@163.com; 2 Key Laboratory of Digital Earth Science, Institute of Remote Sensing and Digital Earth, Chinese Academy of Sciences, Beijing 100094, China; ryc_radi@163.com; 3 School of Public Administration and Mass Media, Beijing Information Science and Technology University, Beijing 100093, China; wangshenghua_sn@163.com

**Keywords:** Wi-Fi indoor positioning, radio map, pedestrian dead reckoning, factor graph optimization

## Abstract

Fingerprinting-based Wi-Fi indoor positioning has great potential for positioning in GPS-denied areas. However, establishing a fingerprinting map (also called a radio map) prior to positioning (site survey) is normally a labor-intensive task. This paper proposes a method for easy site survey without need for any extra hardware. The user can conduct the site survey adopting only a smart phone. The collected inertial-based readings are processed using the pedestrian dead-reckoning algorithms to generate a raw trajectory. Then a factor graph optimization method is proposed to re-estimate the trajectory by adding constraints originated from collected Wi-Fi fingerprints and landmark positions. The proposed method is verified through an experiment in a mall. The mean positioning error is 1.10 m and the maximum error is 2.25 m. This level of positioning accuracy is considered sufficient for radio map generation purposes. A classical baseline algorithm, the k-Nearest Neighbor (kNN) algorithm, is adopted to test the positioning performance of the radio map (RM), which also validates the quality of the constructed RM from the proposed method.

## 1. Introduction

Positioning in GPS-denied areas has been attracting great attention for decades and currently there are many solutions for that. Fingerprinting-based Wi-Fi positioning is one of these solutions with great potential for the following two reasons: (1) No extra hardware is needed. The users only need to carry Wi-Fi-enabled devices, such as smart phones, to locate themselves in indoor environments with abundant Wi-Fi signals. This feature renders the solution suitable for the consumer indoor positioning market as either Wi-Fi-enabled devices or Wi-Fi signals are prevalent in many indoor environments, such as malls and airports; (2) The positioning error is within limited bounds. Unlike inertial-based indoor positioning methods, such as [1,2], the positioning error will not accumulate with time.

The working flow of fingerprinting-based Wi-Fi indoor positioning has two phases: the offline phase (also denoted as site survey phase) and the online phase (also denoted as positioning phase). A fingerprint herein is defined as the received signal strength indication (RSSI) from different Wi-Fi access points (APs) at a fixed location in the environment. Normally, the fingerprint is a vector because there are RSSI from more than one AP. The site survey phase is essentially to associate the fingerprints to geometric positions in the environments. After the phase, each fingerprint corresponds to a position and thus a fingerprint radio map (RM) is established. As the RM is normally established offline, it is also called offline phase. The positioning phase is essentially positioning the user with a Wi-Fi-enabled device online. Each time a new fingerprint is collected by the device, it is matched against the fingerprints from the RM and returns a position. The most commonly seen matching method for this is the k-nearest neighbor (kNN) method [3,4].

Despite the aforementioned two advantages (on the positioning phase), the site survey phase is challenging. Normally, it is recognized as slow and labor-intensive tasks because each position in the environment should be associated with a Wi-Fi signal fingerprint. There are many methods for boosting the efficiency of the site survey phase. The authors in [5] propose to establish the RM in a crowd-sourced way. Many users equipped with foot-mounted inertial measurement units (IMUs) walking in the environments while collecting Wi-Fi fingerprints. After an optimization-based offline process, the trajectories are aligned and calibrated. In this method, the goal is to perform more accurate inertial-based positioning and the RM is a byproduct. The authors in [6] put trajectories generated from a smart phone and some fingerprints at known positions (defined as reference points, RPs) into an optimization framework to establish the RM. The authors in [7,8] propose a quick radio fingerprint collection (QRFC) algorithm for sampling the Wi-Fi signals by clicking on the starting and ending points on the map of the indoor environment. Some methods [9,10,11] propose to sparsely collect fingerprints at some known positions and then reconstruct the RM. Herein many reconstruction methods are adopted, such as compressed sensing (CS) [9] theory, randomized least absolute shrinkage and selection operator (LASSO) [10] and Gaussian process (GP) regression [11]. Interpolation-based methods are also adopted for RM reconstruction purposes using some RPs. In [12], the authors adopt linear interpolation by using the minimum and mean value of the RSS at three RPs, while in [13] a weighted average is adopted. Although interpolation can reduce the number of RPs needed, the accuracy of interpolation-based methods rely on the granularity of existing RPs. The generated RM will not be accurate if the granularity of RPs is very low. Some other methods propose to generate the RM analytically. In [14,15], the authors adopt the existing RSS at the RPs to train the parameters of a pre-defined path loss model, which assumes a logarithmic decay from the distance to the APs, and can greatly reduce the number of RPs needed. However, to acquire the RM using the path loss model, the positions of the APs should be known, which is often not available. Especially in many large scale public sites such as malls and stations, where there can be more than a thousand of APs. Moreover, the trained path loss model may vary in different parts of the indoor environment and in different directions. The radiosity model was originally adopted in physics to describe the heat transfer in bodies with different temperatures [16]. It is adopted in [17] by solving for the Wi-Fi signal propagation problem in the presence of obstacles such as walls and doors. Since the RM is calculated analytically, no domain expert intervention is needed, which can greatly reduce the cost of site survey. However, the radiosity model need detailed indoor maps to begin with, including the position & materials of the walls, doors and so on. Ray tracing [18]-based methods also construct the RM analytically. In [19], ray tracing-based method along with the uniform theory of diffraction (UTD) is adopted to predict the RF propagation. The mentioned methods are effective in generating the RM. However, they either need extra hardware (e.g., foot-mounted IMU), extra information (e.g., detailed indoor maps and building materials) or extra surveying process (fingerprints at known positions).

**Figure 1:**
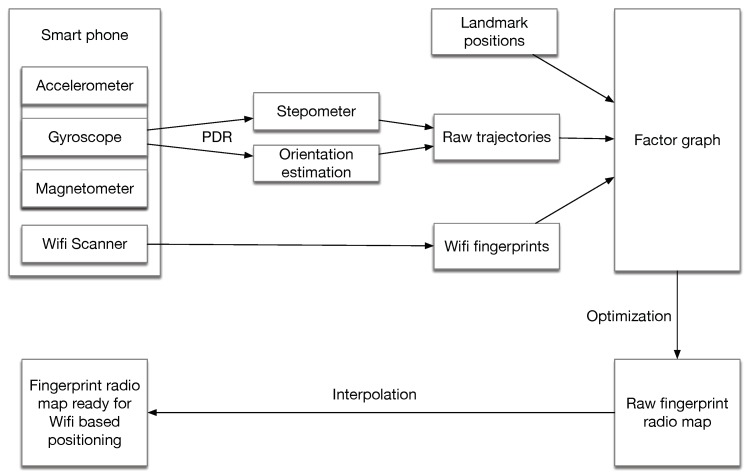
An overall processing flow for the proposed fingerprint radio map establishment.

This paper proposes a method for generating the RM for the site survey phase. As described in Figure 1, the inertial measurements from the smart phones are adopted to generate the trajectories using the pedestrian dead-reckoning (PDR) algorithm. The PDR algorithm mainly solves two problems: (1) how to estimate the phone orientation; (2) how to estimate the step length and detect step occurrence (stepometer). The trajectories from PDR, some landmark positions along with the collected fingerprints are adopted to form a factor graph. The factor graph is generally a graphic representation of an estimation problem for the trajectories. The inertial-generated trajectories can be regarded as an initial guess of the trajectories. By optimizing the graph, a raw RM is established with the estimated positions. After simple linear interpolation, the RM is ready for positioning purposes. Compared to the aforementioned RM generation methods, this method does not need any extra hardware, much additional information (e.g., detailed indoor maps) or extra surveying process. The only extra information is some foreknown landmarks positions, which is commonly fulfilled because many indoor positioning applications needs some reference points to begin with.

Some related work including basics of PDR and factor graph estimation is given in Section 2. Section 3 describes the trajectory generation method based on PDR and Section 4 describes the RM generation method based on factor graph optimization. Real-scenario experiments are carried out in Section 5 to validate the proposed method. Section 6 is the conclusion.

## 2. Related Work

As described in Figure 1, our method relies on trajectories generated from the sensors on the smart phone. This is possible because currently most smart phones have embedded Micro-Electro-Mechanical System (MEMS) accelerometers, gyroscopes and magnetometers. Factor graph-based optimization is widely adopted in simultaneous localization and mapping (SLAM) for finding the poses which best satisfies the measurements. In this section, we give a brief overview of the PDR algorithm and factor graph-based optimization.

### 2.1. Pedestrian Dead-Reckoning

The PDR algorithm consists of two aspects: orientation estimation and stepometer estimation [20]. Herein stepometer estimation includes step occurrence detection and step length estimation. For orientation estimation, the simplest form of the PDR algorithm is to use a simple Kalman filter for updating the orientation [21]. Classical step occurrence detection methods include peak detection [22] and cross-zero detection [23]. These methods assume that the peaks of the projected acceleration or the “cross-zero” points corresponds to new steps. Classical step length estimation method adopts a parametric model [24] and the parameter differs across persons. Noting that the PDR algorithm here avoids double integration of the accelerations to reduce quicker accumulation of acceleration inertial drifts. However, as both step occurrence detection and step length estimation are based on heuristic assumptions, additional positioning errors are introduced, e.g., inaccurate step counts and inaccurate step length estimation. Therefore, PDR is often a part of hybrid positioning solutions rather than a standalone one.

### 2.2. Factor Graph-Based Optimization

Factor graph model is in essence a graphic representation for estimation problems and it is widely adopted in SLAM. It is an alternative for sequential Bayesian estimation other than filter-based frameworks, e.g., Kalman filter and particle filter. In filter-based frameworks, the estimation problem is to recursively estimate a posterior and is suitable for online processing [25]. For factor graph optimization, the estimation problem is transformed to minimizing an error energy function. The error energy function is normally in a least square form and can represent the errors between the variables (needs to be estimated) derived “measurements” and the actual measurements. Unlike the filter-based framework, factor graph optimization estimates the variables in a batch and thus is processed offline.

Factor graph optimization generally consists of two steps: construct the factor graph (equivalent to form the error energy function) and optimize the graph. The first step differs across methods and implementations while the second step has general frameworks. It can be regarded as a general least square optimization (LSO) problem, which can be solved via iterative local linearizations using the Gauss-Newton or Levenberg-Marquardt [26] algorithms. By adopting the sparse nature of the graphs, the computational complexity can be greatly lowered using techniques such as Cholesky decompositions [27] and so on. As there already exists some mature and open-sourced methods for solving such LSO problem such as g2o [28] and ceres-solver [29], we focus on constructing the factor graph in this paper. In our implementation, we use the ceres-solver for the LSO problem.

## 3. Trajectory Generation Based on PDR

As aforementioned, the PDR algorithm consists of orientation estimation and stepometer estimation. In this paper a gradient descent-based orientation estimation algorithm is adopted, which adopts the measured magnetic direction and acceleration to compensate for the orientation error derived from integration over only angular rates. As for the stepometer estimation, we adopt the classical peak detection and heuristic step length estimation algorithms. With the estimated step length $L_{t}$ and the heading $\theta_{t}$ at the current step at *t*, we can derive the current position $\left(x_{t},y_{t}\right)$ from the previous step $\left(x_{t-1},y_{t-1}\right)$

(1) $$\begin{array}{c} x_{t}=x_{t-1}+L_{t}cos\left(\theta_{t}\right)\\ y_{t}=y_{t-1}+L_{t}sin\left(\theta_{t}\right) \end{array}$$

**Figure 2:**
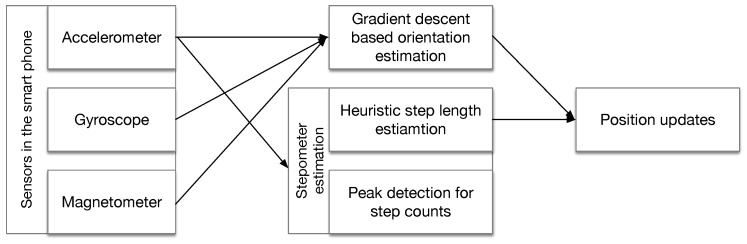
The processing flow for PDR in this paper.

Noting that as Equation (1) shows, the positioning error has accumulative nature because the next position is dependent on the previous one. The processing flow of the PDR algorithm adopted in our paper in Figure 2.

### 3.1. Orientation Estimation

The gradient descent-based orientation estimation method was proposed by [30], which was originally adopted for an inertial-based human motion tracking system. Here we give a brief overview of how it is adopted for orientation estimation in PDR. The three steps are as follows:

1. Orientation updates based on angular rate measurements.
  To avoid singularities, a quaternion representation is adopted here. The attitude of the phone can be updated according to the angular rate measurements in Equation (2)
  (2) $$\begin{array}{c} {}^{S}\omega =\left[0,\omega_{x},\omega_{y},\omega_{z}\right]\\ {}_{N}^{S}\dot{q}=0.5{}_{N}^{S}\bar{q}⊗{}^{S}\omega \end{array}$$
  here $\left(\omega_{x},\omega_{y},\omega_{z}\right)$ is the measured angular rate from the gyroscope. The superscript *S* denotes the sensor frame and the subscript *N* denotes the navigation frame. ${}_{N}^{S}\dot{q}$ denotes the quaternion derivative describing change rate of the navigation frame relative to the sensor frame. ⊗ is the quaternion product operation and the bar of $\bar{q}$ denotes the normalization operation of a quaternion. For discrete time implementation, it is
  (3) $$\begin{array}{c} {}_{N}^{S}{\dot{q}}_{t}=0.5_{N}^{S}{\bar{q}}_{est,t-1}{⊗}^{S}\omega \\ {}_{N}^{S}q_{est,t}{=}_{N}^{S}{\bar{q}}_{est,t-1}{+}_{N}^{S}{\dot{q}}_{t}\delta t \end{array}$$
  δt is the sampling interval of the gyroscope and we set it to 0.01 s in our implementation. Using Equation (3), the quaternion of the attitude can be updated. However, the error of the attitude is accumulative due to gyroscope drifts.
2. Forming the error function. To estimate the attitude more accurately, the measurements from the magnetometer and the accelerometer is adopted. An error function f(.) is formed for such purposes in Equation (4).
  (4) $$f\left(.\right){=}_{N}^{S}{\bar{q}}^{*}{⊗}^{N}\bar{M}{⊗}_{N}^{S}\bar{q}{-}^{S}\bar{M}$$
  * is the quaternion adjoint operation. The error function denotes the differences of normalized measurements (accelerometer measurements or magnetometer measurements) between representation in the sensor frame (${}^{S}\bar{M}$) and derived representation in the sensor frame (${}_{N}^{S}{\bar{q}}^{*}{⊗}^{N}\bar{M}{⊗}_{N}^{S}\bar{q}$) from the representation in the navigation frame (${}^{N}\bar{M}$). Noting that ${}^{S}\bar{M}$ and ${}^{N}\bar{M}$ has a normalized quaternion representation.
  Specifically, for the accelerometer measurements, ${}^{S}\bar{M}$ and ${}^{N}\bar{M}$ is replaced by ${}^{S}\bar{a}$ and ${}^{N}\bar{g}$ respectively, where
  (5) $$\begin{array}{c} {}^{S}\bar{a}=\left(0,a_{x},a_{y},a_{z}\right)\\ {}^{N}\bar{g}=\left(0,0,0,1\right) \end{array}$$
  here the $\left(a_{x},a_{y},a_{z}\right)$ is the normalized readings from the accelerometer. Noting that there is only one non-zero element in ${}^{N}\bar{g}$, because when the phone is stationary or quasi-stationary, the only accelerometer should be the z-axis gravity.
  For the magnetometer measurements, ${}^{S}\bar{M}$ and ${}^{N}\bar{M}$ is replaced by ${}^{S}\bar{m}$ and ${}^{N}\bar{b}$ respectively, where
  (6) $$\begin{array}{c} {}^{S}\bar{m}=\left(0,m_{x},m_{y},m_{z}\right)\\ {}^{N}\bar{b}=\left(0,b_{x},0,b_{z}\right) \end{array}$$
  here the $\left(m_{x},m_{y},m_{z}\right)$ is the normalized reading from the magnetometer. In the navigation frame, the magnetic field can be considered only has horizontal component and vertical component, so ${}^{N}\bar{b}$ only has two component. With the magnetometer readings and the accelerometer readings, the combined error function can be written as
  (7) $$f_{g,b}=\left[\begin{array}{c} f_{g}\left(.\right)\\ f_{b}\left(.\right) \end{array}\right]$$
  where $f_{g}\left(.\right)$ is formed by replacing ${}^{S}\bar{M}$ and ${}^{N}\bar{M}$ in Equations (4) and (5), and $f_{b}\left(.\right)$ is formed by replacing ${}^{S}\bar{M}$ and ${}^{N}\bar{M}$ in Equations (4)–(6). Then we have
  (8) $$f_{g}\left(.\right)=\left[\begin{array}{c} 2\left(q_{2}q_{4}-q_{1}q_{3}\right)-a_{x}\\ 2\left(q_{1}q_{2}-q_{3}q_{4}\right)-a_{y}\\ 2\left(0.5-q_{2}^{2}-q_{3}^{2}\right)-a_{z} \end{array}\right]$$
  and
  (9) $$f_{b}\left(.\right)=\left[\begin{array}{c} 2b_{x}\left(0.5-q_{3}^{2}-q_{4}^{2}\right)-2b_{z}\left(q_{2}q_{4}-q_{1}q_{3}\right)-m_{x}\\ 2b_{x}\left(q_{2}q_{3}-q_{1}q_{4}\right)+2b_{z}\left(q_{1}q_{2}+q_{3}q_{4}\right)-m_{y}\\ 2b_{x}\left(q_{1}q_{3}+q_{2}q_{4}\right)+2b_{z}\left(0.5-q_{2}^{2}-q_{3}^{2}\right)-m_{z} \end{array}\right]$$
  $q_{1},q_{2},q_{3},q_{4}$ are the components of the quaternion ${}_{N}^{S}{\bar{q}}_{est,t-1}$.
3. Gradient descent for orientation estimation. To minimize the error function, the gradient descent method is adopted. Noting that here we only update the current estimation per time sample according to
  (10) $${}_{N}^{S}q_{\nabla ,t}{=}_{N}^{S}{\bar{q}}_{est,t-1}-\gamma_{t}\frac{\nabla f_{g,b}\left(.\right)}{∥\nabla f_{g,b}\left(.\right)∥}$$
  $\nabla f_{g.b}\left(\right)$ is the gradient of the error function $f_{g,b}\left(.\right)$ and $\gamma_{t}$ is a proper scale controlling descending velocity. Same as [30], $\gamma_{t}$ is set to
  (11) $$\gamma_{t}=∥{}_{N}^{S}\dot{q}∥\delta t$$
  where δt is the sampling interval.
  Combined with Equation (3), the attitude update process can be written as
  (12) $$\begin{array}{c} {}_{N}^{S}q_{t,est}{=}_{N}^{S}{\bar{q}}_{est,t-1}{+}_{N}^{S}{\dot{q}}_{est,t-1}\delta t\\ {}_{N}^{S}{\dot{q}}_{est,t-1}=0.5_{N}^{S}{\bar{q}}_{est,t-1}{⊗}^{S}\omega +\beta \frac{\nabla f_{g,b}\left(.\right)}{∥\nabla f_{g,b}\left(.\right)∥} \end{array}$$
  where the coefficient β is
  (13) $$\beta =\sqrt{\frac{3}{4}\omega_{err,max}}$$
  $\omega_{err,max}$ is the maximum gyroscope measurement error. The derivation of β is explained in detail in [30] and is directly used in our paper. Then from the attitude quaternion, we can solve for the heading angle as the orientation $\theta_{t}$.

### 3.2. Stepometer Estimation

For step occurrence detection we adopt the classical peak detection method. For the step length estimation, we adopt a heuristic parametric model in [24] as

(14) $$L_{t}=K^{4}\sqrt{a_{max}-a_{min}}$$

where $a_{max}$ and $a_{min}$ are the maximum and minimum acceleration during the step. *K* is a parameter which should be different across user to user. Here we set is as a constant 0.5. Noting that the step length estimation error is one of the error sources of PDR-based position tracking. The trajectories generated from PDR will later be re-estimated using Wi-Fi fingerprints and some pre-known landmark positions.

**Figure 3:**
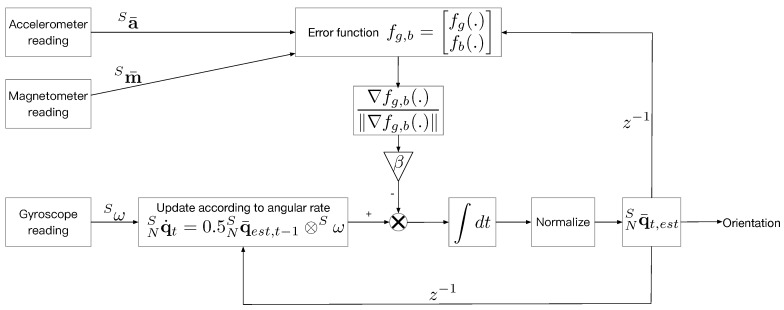
The processing of orientation estimation using readings from the accelerometer, magnetometer and gyroscope.

To sum up, the processing flow of orientation estimation is shown in Figure 3, where $z^{-1}$ denotes one sample interval time back.

## 4. Factor Graph Optimization for RM Generation

**Figure 4:**
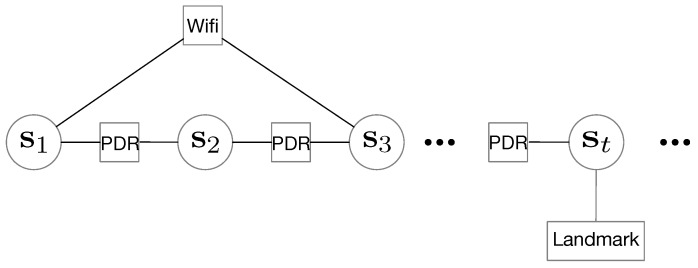
An illustration of the factor graph for RM generation. There are three types of edges: Wi-Fi-based edges, PDR-based edges and landmark-based edges.

The factor graph for RM generation in our method is shown in Figure 4. The variables in the circles denotes the poses at different times, which needs to be estimated. In our implementation, a pose consists of three components

(15) $$s_{t}=\left[\begin{array}{c} x_{t}\\ y_{t}\\ \theta_{t} \end{array}\right]$$

where $x_{t}$ and $y_{t}$ are the horizontal positions and $\theta_{t}$ is the heading. From the factor graph, both Wi-Fi-based edges and PDR-based edges denote constraints between two poses, while the landmark-based edge denotes a constraints to a single pose. An error energy function can be drawn from the factor graph

(16) $$F\left(s_{1:t}\right)=F_{PDR}\left(s_{1:t}\right)+F_{Wifi}\left(s_{1:t}\right)+F_{landmark}\left(s_{1:t}\right)$$

All the three sources of error energy have a quadratic form

(17) $$F_{\left(.\right)}\left(.\right)=e_{\left(.\right)}{\left(.\right)}^{T}\Omega_{\left(.\right)}e_{\left(.\right)}\left(.\right)$$

where $e_{\left(.\right)}\left(.\right)$ denotes the error between the actual measurements and measurements derived from the poses to be estimated. $\Omega_{\left(.\right)}$ denotes the information matrix of the measurements. By minimizing $\left(s_{1:t}\right)$, the maximum likelihood estimation of poses can be acquired. As the minimization process can be regarded as a general LSO problem, we adopt the ceres-solver [29] for solving the problem. Here we focus on how to form the different sources of error energy functions.

After the optimization, the sequences of optimal poses are acquired. With these poses and the collected fingerprints, a raw RM can be established with associated fingerprints and geometric positions. We use an interpolation and extrapolation method in [31] to transform the raw RM into a grid-based one and make it ready for the online positioning phase.

### 4.1. PDR-Based Error Energy

The PDR algorithm can provide inertial generated trajectories. These trajectories (time sequences of poses) can be regarded as the initial values of the poses to be estimated or optimized. The PDR algorithm can also provide pose changes between adjacent steps. The pose change from the PDR algorithm is $u_{t}^{PDR}$,

(18) $$u_{t}^{PDR}=\left[\begin{array}{c} L_{t}^{PDR}\\ \delta \theta_{t}^{PDR} \end{array}\right]$$

where $L_{t}^{PDR}$ is the step length from PDR and $\delta \theta_{t}^{PDR}$ is the heading change from PDR. The pose change derived from the pose variables s is

(19) $$u_{t}^{s}=\left[\begin{array}{c} \sqrt{{\left(x_{t}^{s}-x_{t-1}^{s}\right)}^{2}+{\left(y_{t}^{s}-y_{t-1}^{s}\right)}^{2}}\\ \theta_{t}^{s}-\theta_{t-1}^{s} \end{array}\right]$$

Then the PDR-based error energy is

(20) $$F_{PDR}\left(s_{1:t}\right)=\sum_{t}e_{PDR}{\left(s_{t-1},s_{t},u_{t}^{PDR}\right)}^{T}\Omega_{t,PDR}e_{PDR}\left(s_{t-1},s_{t},u_{t}^{PDR}\right)$$

where the error $e_{PDR}\left(.\right)$ is dependent on adjacent pose variable $s_{t-1}$, $s_{t}$ and the pose change from the PDR process $u_{t}^{PDR}$.

(21) $$e_{PDR}\left(s_{t-1},s_{t},u_{t}^{PDR}\right)=u_{t}^{s}-u_{t}^{PDR}$$

The information matrix for the PDR-based error has the form

(22) $$\Omega_{t,PDR}=\left[\begin{array}{cc} Info(L_{t}) & 0\\ 0 & Info(\delta \theta_{t}) \end{array}\right]$$

where $Info(L_{t})$ and $Info(\delta \theta_{t})$ should be the reciprocal of the variance of PDR-based step length and heading change estimation respectively,

(23) $$\begin{array}{c} Info\left(L_{t}\right)=\frac{1}{\sigma_{L_{t}}^{2}}\\ Info\left(\delta \theta_{t}\right)=\frac{1}{\sigma_{\delta \theta_{t}}^{2}} \end{array}$$

In our implementation, $Info(L_{t})$ and $Info(\delta \theta_{t})$ are set to 20 and 500 respectively. If no other types of error energy are available, the minimum of $e_{PDR}\left(.\right)$ should be zero, when $u_{t}^{s}$ equals $u_{t}^{PDR}$ at every step. In this situation, the optimal pose estimations are the poses in the inertial generated trajectories from PDR.

### 4.2. Wi-Fi-Based Error Energy

**Figure 5:**
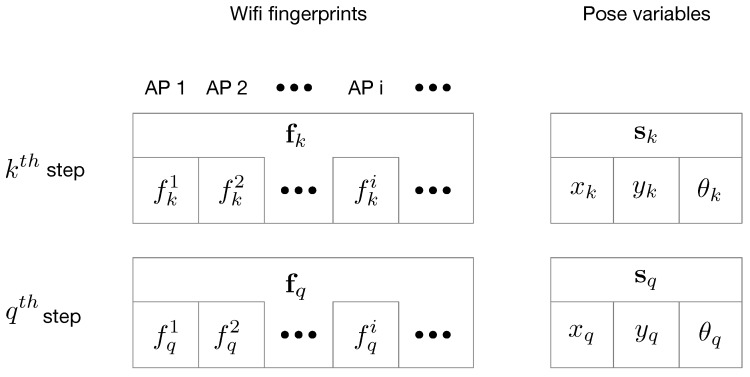
An illustration for the correspondences between the fingerprints and the pose variables.

**Figure 6:**
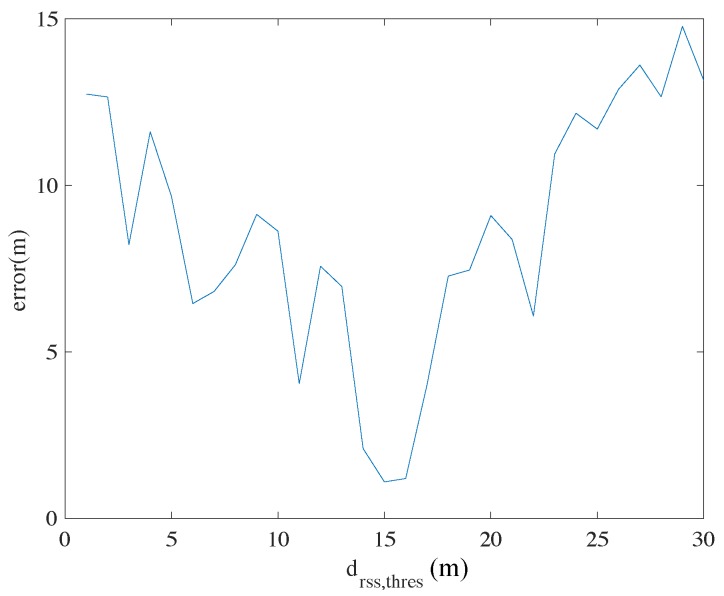
The mean positioning errors of the proposed method according to different values of $d_{thres,rss}$.

Wi-Fi-based error energy is formed using the following two steps:

1. Find the distance between two fingerprints at the $k^{th}$ and $q^{th}$ step.
  In Wi-Fi-based fingerprinting methods, a common assumption is often held true: if two fingerprints are with vicinity in signal space, then the positions where the fingerprints are collected are with vicinity in the coordinate space. In our method, we also find the correspondences of positions by solving for the vicinities in signal space. We compare two arbitrary Wi-Fi fingerprints $f_{k}$ and $f_{q}$ which are collected at the $k^{th}$ step and the $q^{th}$ step (k≠q). Then we define their distance in signal space like this using a metric similar to [6]
  (24) $$d\left(f_{k},f_{q}\right)=\sqrt{\frac{\sum_{i}{\left(f_{k}^{i}-f_{q}^{i}\right)}^{2}}{N}}$$
  where the superscript *i* on $f_{k}^{i}$ denotes the RSS from the $i^{th}$ AP in the vector $f_{k}$, and *N* is the number of AP in the vector. The correspondences of the fingerprints and the pose variables are shown in Figure 5.
2. Find the error $e_{Wifi}\left(s_{k},s_{q},f_{k},f_{q}\right)$ according to the distance in the signal space.
  In our implementation, if two fingerprints’ distance is less than a pre-defined threshold, the distances of the corresponding poses should be within a threshold (with vicinity). Then we define the Wi-Fi-based error like this
  (25) $$e_{Wifi}\left(s_{k},s_{q},f_{k},f_{q}\right)=\left\{\begin{array}{cc} d(s_{k},s_{q}), & if\;d\left(f_{k},f_{q}\right)<d_{thres,rss}\\ 0, & if\;d\left(f_{k},f_{q}\right)>d_{thres,rss} \end{array}\right.$$
  where $d(s_{k},s_{q})$ means the Euclidian distance between the horizontal positions of $\left(x_{k},y_{k}\right)$ and $\left(x_{q},y_{q}\right)$ calculated from the pose variables $s_{k}$ and $s_{q}$. Figure 6 shows the relationships between the mean positioning errors and different values of $d_{thres,rss}$ in the experiment (details of the experiment will be described in Section 5). We can see that in our implementation, the mean error reaches the lowest around $d_{thres,rss}=15$. This value is thus adopted for creating Wi-Fi-based constraints.

Then the Wi-Fi-based error energy is

(26) $$F_{Wifi}\left(s_{1:t}\right)=\sum_{k,q}e_{Wifi}^{2}\left(s_{k},s_{q},f_{k},f_{q}\right)\Omega_{Wifi}$$

where *k* and *q* are two arbitrary step index from 1:*t* and k≠q. Noting that the error is a constant zero when $d\left(f_{k},f_{q}\right)>d_{thres,rss}$ as shown in Equation (25) and will not contribute to Equation (26). In our implementation, we only include the errors when $d\left(f_{k},f_{q}\right)<d_{thres,rss}$. This will significantly reduce the number of constraints and is favorable in terms of computational cost for the optimization process later. Here the error $e_{Wifi}\left(.\right)$, as well as $\Omega_{Wifi}$ are both scalars.

### 4.3. Landmark-Based Error Energy

Assuming we have a pre-defined landmark position $p_{lm,j}=\left(x_{lm,j},y_{lm,j}\right)$ and we have already established the correspondence to the pose variable $s_{j}$ at the $j^{th}$ step. This can be easily done by ask the user to press a button on the smart phone when walked to the landmark. Then the landmark-based error is

(27) $$e_{landmark}\left(u_{j},p_{lm,j}\right)=\sqrt{{\left(x_{lm,j}-x_{j}\right)}^{2}+{\left(y_{lm,j}-y_{j}\right)}^{2}}$$

where $\left(x_{j},y_{j}\right)$ is the horizontal positions taken from the pose variable $u_{j}$ and the error $e_{landmark}\left(u_{j},p_{lm,j}\right)$ is also a scaler. Then the landmark-based error energy function is

(28) $$F_{landmark}\left(s_{1:t}\right)=\sum_{j}e_{landmark}^{2}\left(u_{j},p_{lm,j}\right)\Omega_{landmark}$$

where the sum is over all recorded landmarks by the users and $\Omega_{landmark}$ should be a relatively large number, because it is believed the landmark positions are accurate.

## 5. Experiment

### 5.1. Experimental Setup

In our experiment, the Huawei Mate 9 smart phone is adopted. An Android application is developed for collecting data. The application has three main functions.

1. Generate the raw trajectories based on the PDR algorithm. The sampling rate of the accelerometer, gyroscope and magnetometer sensors in the phone is set to 100 Hz. The readings from these sensors can be processed in real time and can generate inertial-based raw trajectories. These poses of the trajectories with timestamps of the phone’s system time are saved as file.
2. Collect Wi-Fi-based fingerprints. The Wi-Fi scanner on the phone is set to continuous scan mode with scanning interval of 1 s. However, the actual scanning interval can only reach about 2.5 s (due to system limitations). The fingerprints along with their collecting time are also saved as a file.
3. Record landmarks by pressing the landmark button. When walks to a pre-defined landmark, the user can press the buttons on the phone to record the time and landmark number.

All the types of data are saved as file on the phone with its collecting time. Afterwards, the data can be synchronized and processed offline to generate the RM. We assess the accuracy of the generated RM by assessing the accuracy of the positions where the fingerprints are collected (or the trajectories). In our implementation, we record as many landmarks as possible. In this way, a part of the landmark recordings can be taken to generate the RM, the left part as ground truth positions to assess the accuracy of the optimized poses (trajectories). In the experiment, the positions of the landmarks are measured with a total station with sufficient accuracy.

### 5.2. Raw Trajectories Based on PDR

**Figure 7:**
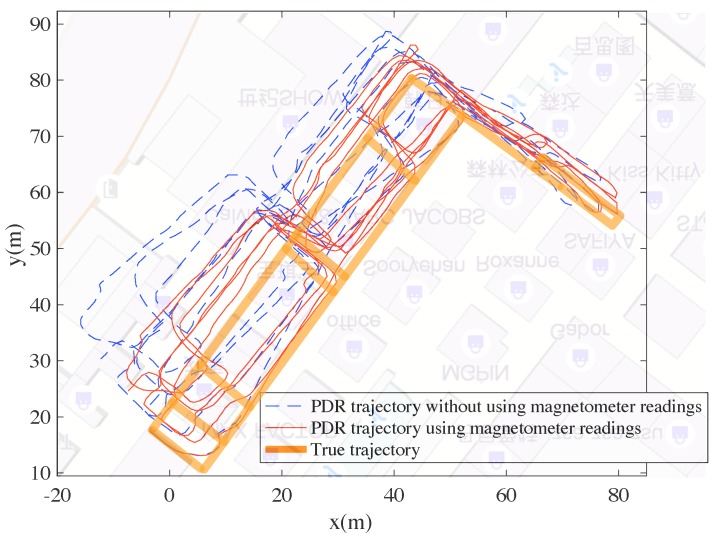
The PDR trajectories with and without magnetometer readings.

In Figure 7, a user walks in a mall with a hand-held smart phone. The generated trajectories from the PDR algorithm are shown. It is obvious that the estimated trajectory with magnetometer readings is more consistent with the true trajectory than the one without magnetometer readings. This is because the orientation estimation is much more accurate using magnetometer readings. This validates the orientation estimation method adopted in this paper. However, even adopting magnetometer readings, the PDR trajectory still cannot reach sufficient accuracy (the trajectory does not fit the floor plan and the true trajectory well). Other information is needed to re-estimate the trajectory to improve accuracy.

### 5.3. Factor Graph Optimization Results

The PDR trajectory can be re-estimated using factor graph optimization if other types of information are available. Here we present the optimized trajectory using PDR trajectory and

1. Wi-Fi fingerprints
2. landmark positions
3. Wi-Fi fingerprints and landmark positions

Respectively. In this experiment, we have set 18 different landmarks with known positions. The user has walked to these landmarks for 88 times, so that there are 88 landmark recordings in total. We only take 20 of them for factor graph optimization and the left 68 recordings as ground truth positions to verify the accuracy.

#### 5.3.1. Results for Fusing PDR Trajectory and Wi-Fi-Based Constraints

**Figure 8:**
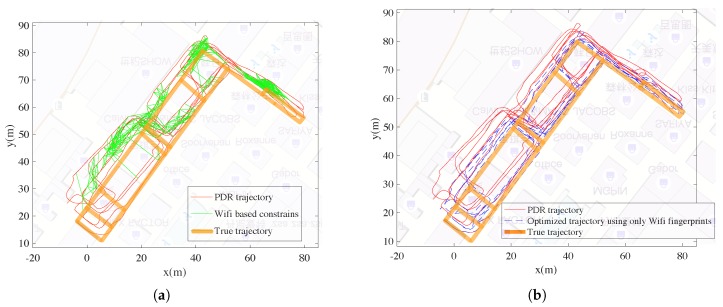
Results for fusing PDR trajectory and Wi-Fi fingerprints. (**a**) Wi-Fi-based constraints. (**b**) Optimized trajectory using Wi-Fi-based constraints.

Figure 8a shows the constraints of poses derived from Wi-Fi fingerprints. If two poses are connected with a green line, it means the fingerprints collected at the two poses are with vicinity in the signal space (Wi-Fi distance less than a threshold). In this case, the two poses should be also with vicinity and the term of the error energy should grow with the distances between the two poses. Noting that there are some false alarm constraints and can be regarded as outlier constraints. Figure 8b shows the optimized trajectory using Wi-Fi fingerprints, which is more accurate than the original PDR trajectory.

#### 5.3.2. Results for Fusing PDR Trajectory and Landmark Based Constraints

**Figure 9:**
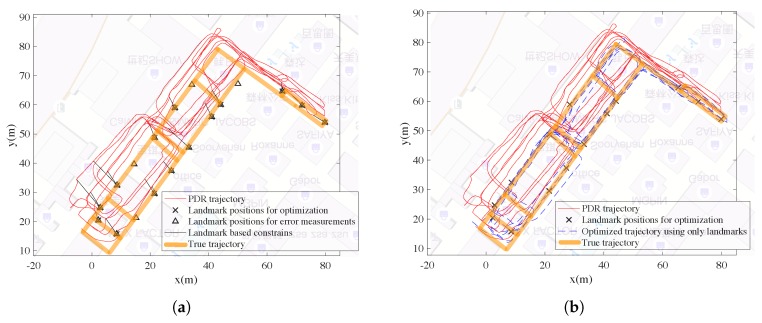
Results for fusing PDR trajectory and landmark positions. (**a**) Landmark based constraints. (**b**) Optimized trajectory using landmark constraints.

As mentioned before, the user has walked to the landmark for 88 times. However, we only randomly choose 20 of them as landmark constraints for optimization (training data), and the left is adopted to test the positioning accuracy (test data). As shown in Figure 9a, the landmark positions for training are black crosses and the test ones are black triangles. The black lines denote the constraints between the poses in the trajectory and the landmarks. Here there are only 20 constraints. The landmark positions for training can be regarded as direct observation of the poses’ positions. After optimization adopting the landmark positions, the trajectory is shown in Figure 9b.

#### 5.3.3. Results for Fusing PDR Trajectory, Wi-Fi-Based Constraints and Landmark-Based Constraints

**Figure 10:**
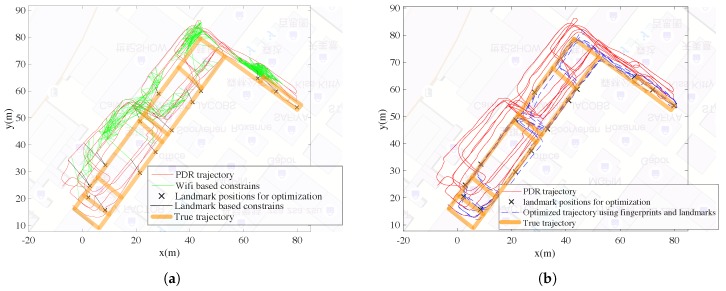
Results for fusing PDR trajectory, Wifi fingerprints and landmark positions. (**a**) Wifi based and landmark based constraints. (**b**) Optimized trajectory.

The Wi-Fi-based constraints and the landmark-based constraints are shown together in Figure 10a, and the optimized trajectory are shown in Figure 10b. As mentioned, the positioning errors are measured according to the left 68 test landmark positions which are not adopted for optimization.

**Figure 11:**
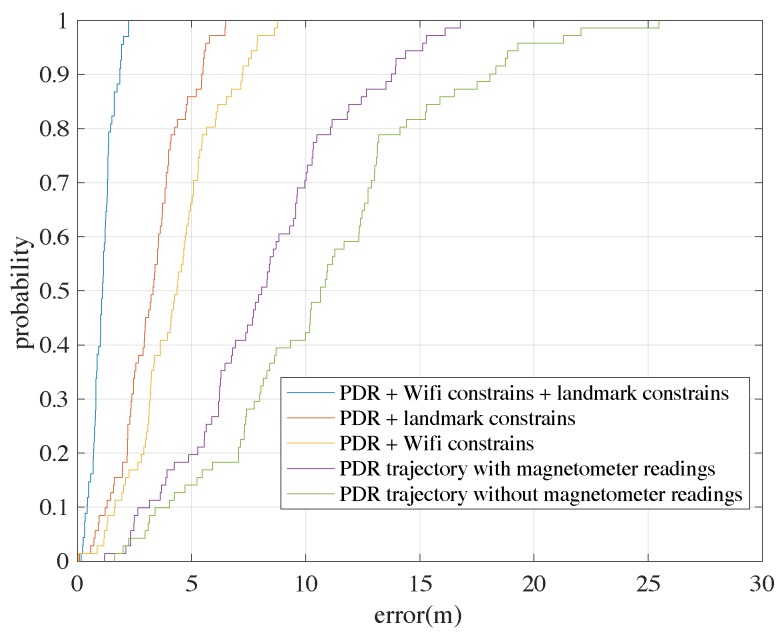
The CDFs of positioning errors at the test landmarks.

Figure 11 gives the cumulative density functions (CDFs) of the positioning errors with different trajectories. We can see the best performance is the result with all types of information including magnetometer readings, PDR results, Wi-Fi constraints and landmark positions. With all the information, the mean positioning error is about 1.10 m and the maximum error is about 2.25 m, which is considered sufficient for RM generation purposes.

Table 1 gives some statistics of the positioning errors using different types of constraints. For pure PDR results, adopting magnetometer readings as suggested in Section 3.1 can help improve the accuracy of the generated trajectory. Noting that the mean error can decrease from 11.35 m to 8.67 m. We can also see that either adopting Wi-Fi-based constraints or landmark based constraints can contribute to improve the positioning accuracy. The mean error of the optimized trajectory adopting all types of constraints can be decreased to about 1.10 m. In addition, the maximum error for the trajectory is only 2.25 m, which shows robustness for position estimation. The errors of the estimated trajectory are sufficient for RM generation purposes.

### 5.4. Wi-Fi-Based Positioning Results Adopting the Generated RM

**Figure 12:**
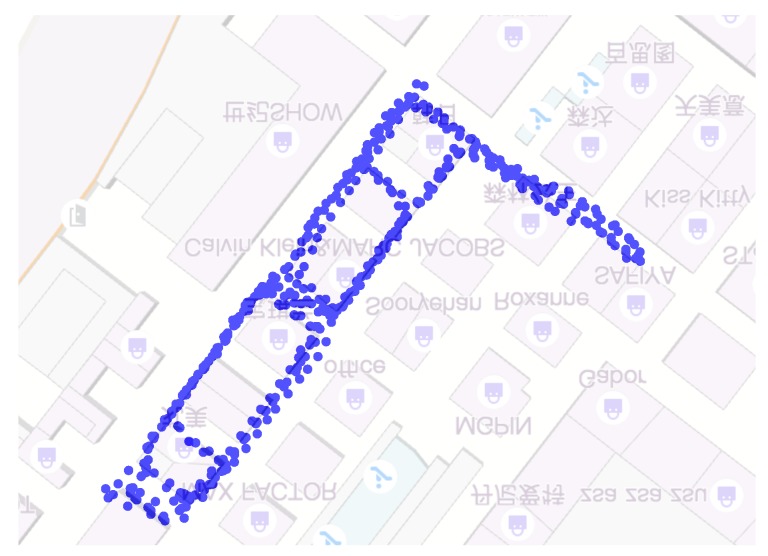
The interpolated positions where fingerprints are collected.

After the graph optimization process, a series of positions (a trajectory) can be estimated. According to the timestamps of the positions and the timestamps of the fingerprints, a simple linear interpolation is made to solve for the positions where the RPs are. After interpolation, the positions of the RPs are available are shown in Figure 12. Noting that we only take one fingerprint at each location for factor graph creation purposes. The final RM includes all fingerprints collected.

**Figure 13:**
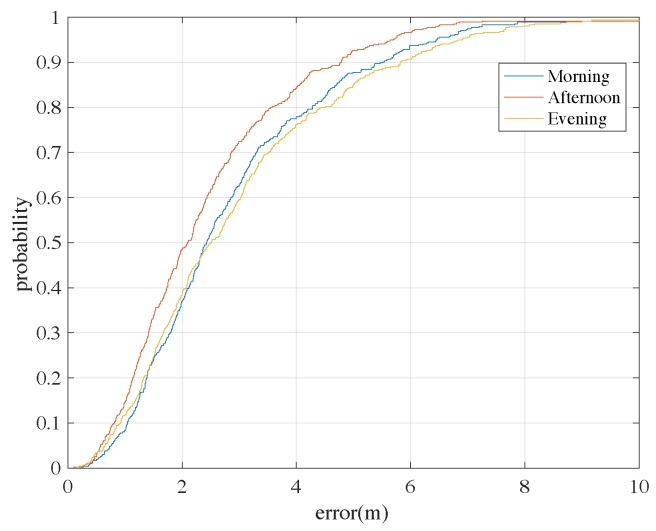
The CDFs of Wi-Fi-based positioning errors adopting the constructed RM at different times of a day using the classical baseline positioning algorithm: the kNN algorithm.

These fingerprints are directly adopted for Wi-Fi-based positioning using the classical kNN algorithm as the baseline approach. Experiments are carried out to show the performance of Wi-Fi-based positioning adopting the constructed RM. According to Figure 13, the CDFs of positioning errors at different times in the day are shown. We can see that about 90% of the errors are less than 5 m for the test in the afternoon (when the RM data are collected). Although in the morning and in the evening the positioning performances are worse, the accuracies are still considered enough for many commercial indoor positioning cases, and can demonstrate the good quality of the constructed RM.

**Figure 14:**
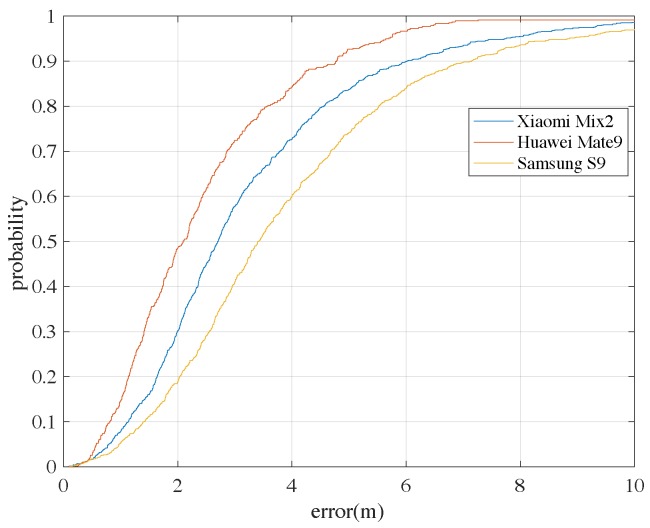
The CDFs of Wi-Fi-based positioning errors adopting the constructed RM with 3 different types of phones. (also using the classical kNN baseline algorithm).

Another experiment is carried out to test how the different types of devices can affect the positioning performance. Here we have shown the positioning errors for 3 different devices adopting the RM (constructed from Huawei Mate 9) in Figure 14. Not surprisingly, the positioning performance of the Xiaomi Mix2 and Samsung S9 is not as good as the Huawei Mate 9, because their measurements of RSS are different. However, this shows that heterogeneous devices do have a large impact on the positioning performance. An easy way for solving the problem is to carry different devices and construct different versions of RMs. The detailed issues of the heterogeneous devices are considered as another topic and will be studied in the future.

## 6. Conclusions

A method for generating Wi-Fi-based RM is proposed in this paper. The method can work without any extra hardware such as an IMU, much additional information such as a detailed indoor map, and any extra surveying process such as collecting Wi-Fi fingerprints at known positions. The user only needs a smart phone to collect inertial-based readings (from the inertial sensors in the phone), Wi-Fi fingerprints and landmark positions. The inertial-based readings are adopted to generate a PDR-based raw trajectory. Then the factor graph optimization method is adopted to re-estimate the trajectory using constraints provided by Wi-Fi fingerprints and landmark positions. An experiment is carried out in a mall with a smart phone for data collection. The results have shown that both Wi-Fi-based constraints and landmark-based constraints can contribute to accurate position estimations in the RM. The accuracy of the optimized trajectory using both Wi-Fi fingerprints and landmark positions can reach a mean error of 1.10m, which is considered sufficient for RM generation purposes. Then the constructed RM can be adopted for Wi-Fi-based positioning with satisfactory accuracies.

## Figures and Tables

**Table 1:** Error statics at the test landmarks for different trajectories.

Trajectroy	Mean Error (m)	Maximum Error (m)	Median Error (m)
PDR trajectory without magnetometer readings	11.35	25.47	10.64
PDR trajectory with magnetometer readings	8.67	15.12	8.08
PDR + Wifi constraints	4.35	7.87	4.36
PDR + landmark constraints	3.12	5.56	3.32
PDR + Wifi constrains + landmark constraints	1.10	2.25	1.09

## Abbreviations

The following abbreviations are used in this manuscript:

RSSI	received signal strength indication
APs	access points
RPs	reference points
RM	radio map
kNN	k-nearest neighbor
IMUs	inertial measurement units
CS	compressed sensing
LASSO	randomized least absolute shrinkage and selection operator
GP	gaussian process
PDR	pedestrian dead reckoning
MEMS	Micro-Electro-Mechanical System
SLAM	simultaneous localization and mapping
LSO	least square optimization
